# Assisted Reproductive Technology Live Birth Rates Decrease Following Panproctocolectomy in Crohn's Disease: A Case Report

**DOI:** 10.7759/cureus.90597

**Published:** 2025-08-20

**Authors:** Despina Nicolaou, Nicolas Nicolaou, Tulay Karasu

**Affiliations:** 1 Obstetrics and Gynaecology, Addenbrooke's Hospital, Cambridge University Hospitals NHS Foundation Trust, Cambridge, GBR; 2 Ophthalmology, Queen Mary University of London, London, GBR; 3 Department of Obstetrics and Gynaecology, Reproductive Medicine, Cambridge University Hospitals, Cambridge, GBR

**Keywords:** assisted reproductive technology (art) live birth rates, chronic endometritis, inflammation and infertility, panproctocolectomy, recurrent implantation failure (rif), surgical crohn's disease, vitamin b12 deficiency

## Abstract

Women with Crohn's disease (CD) are often affected during their reproductive years. Live birth rates following assisted reproductive technology (ART) in medically managed CD are comparable to those in the general population. However, ART outcomes decline significantly after CD-related surgery, a pattern not observed in ulcerative colitis (UC). We report the case of a 33-year-old woman with longstanding perianal and vulvovaginal CD who underwent a panproctocolectomy with end ileostomy at age 28. Postoperatively, she developed an enterovaginal fistula and recurrent bacterial vaginosis. Despite being in remission, she achieved only one biochemical pregnancy across five ART cycles. Investigations revealed group B Streptococcus vaginal colonisation and chronic endometritis (CE). This case highlights multiple factors that may impair fertility following surgical management of CD, including pelvic adhesions, CE, microbial dysbiosis, immune dysfunction, and micronutrient deficiencies such as vitamin B12. Unlike UC, surgery is not curative in CD and relapse is common. Where possible, major abdominal surgery in CD should be delayed until after childbearing to preserve fertility. A multidisciplinary approach is essential, and preoperative fertility counselling is strongly recommended. Further research and clear clinical guidelines are needed to optimise reproductive outcomes in women undergoing surgery for CD.

## Introduction

In women with Crohn's disease (CD), a chronic inflammatory bowel condition, most cases occur during the reproductive years [1]. When CD is medically managed to remission, live birth rates with assisted reproductive technology (ART) are comparable to those in women without the disease [2]. ART includes procedures such as in vitro fertilisation (IVF) and intracytoplasmic sperm injection (ICSI), where an egg is fertilised with sperm in a laboratory before transfer to the uterus. However, 5.8-25% of patients with refractory CD (resistant to medical management) require surgery, such as panproctocolectomy (removal of the colon, rectum, and anus) with ileal pouch-anal anastomosis (IPAA) or an end ileostomy [1,2]. ART live birth rates are markedly reduced by approximately 49-71% in women with prior CD IPAA surgery, even in remission [2,3]. In contrast, similar surgeries for ulcerative colitis (UC), an inflammatory bowel disease affecting only the colon, do not appear to significantly affect fertility [2]. The mechanisms underlying reduced fertility after CD surgery, unlike UC, remain poorly understood. This case describes a patient with perianal and vulvovaginal Crohn's and prior surgery who underwent five unsuccessful ART cycles. It explores factors contributing to fertility impairment and highlights the importance of multidisciplinary collaboration and preoperative counselling. It also emphasises the need for evidence-based guidelines to support fertility preservation in women undergoing major CD surgery.

## Case presentation

A 33-year-old woman with a 20-year history of transmural CD with colonic and rectal involvement and penetrating (B3) behaviour was referred for ART. Her condition, initially managed with azathioprine, improved with infliximab infusions administered every eight weeks. Later, she developed vulval granulomatous inflammation consistent with metastatic vulvar CD, confirmed by two biopsies. Due to refractory disease, she underwent a total panproctocolectomy with end ileostomy at age 28, with minimal counselling provided on the potential impacts of surgery on fertility. Postoperatively, she continued to report persistent vulvovaginal symptoms, including swelling, dryness, dyspareunia, and intermittent post-coital dysuria. Further biopsies revealed inflammation associated with vulvar intraepithelial neoplasia (VIN) grade 2, prompting referral to gynaecology and Human Papillomavirus (HPV) genotyping. Given prior resistance to immunomodulators, anti-tumor necrosis factor (TNF) therapy, adalimumab was initiated.

**Figure 1:**
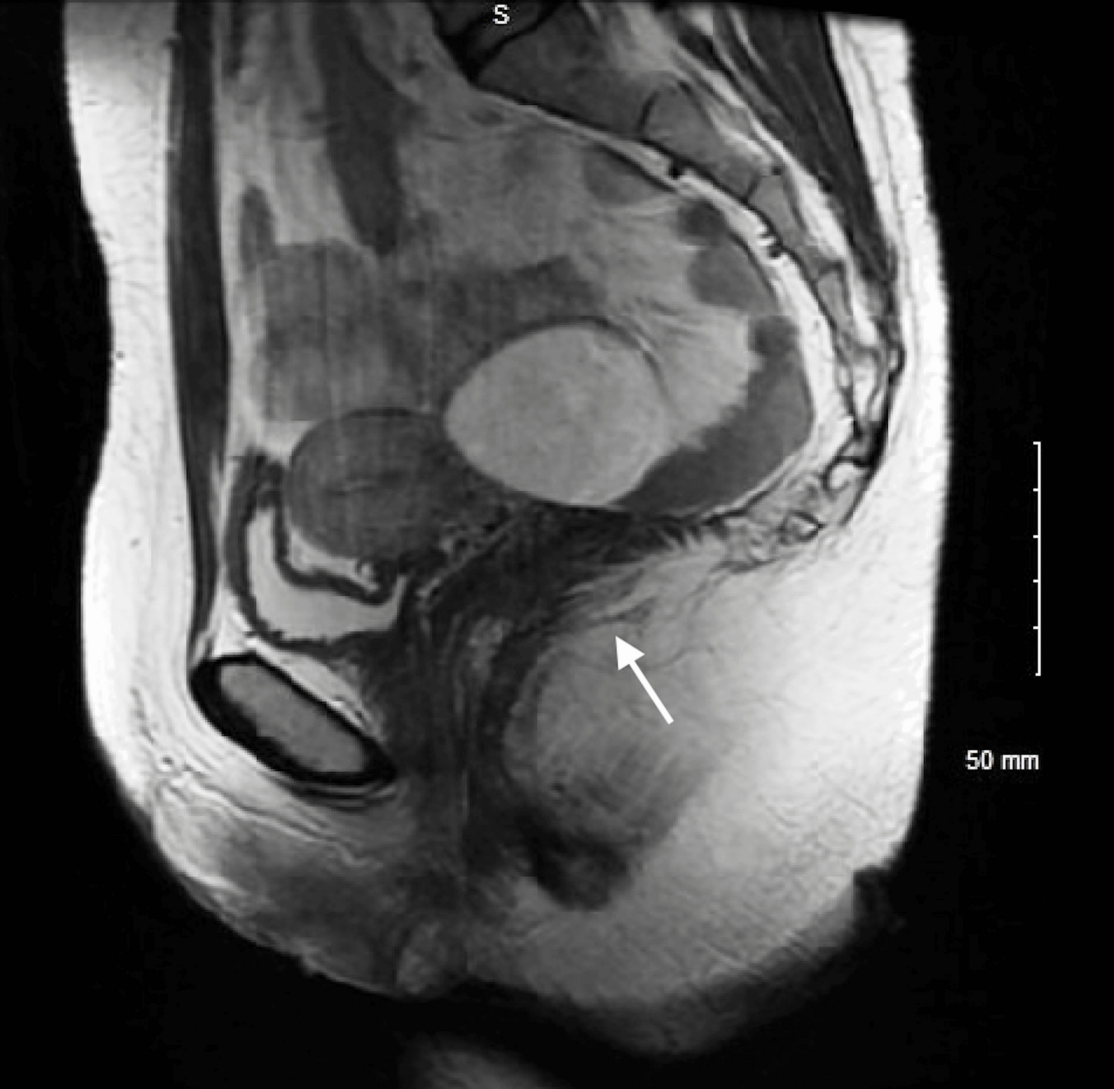
Pelvic magnetic resonance imaging (MRI) post-panproctocolectomy in a 28-year-old woman with Crohn's disease. Sagittal MRI of a female pelvis following panproctocolectomy, showing absent rectum and sigmoid colon. A linear high-signal tract extending posteriorly from the vaginal vault indicates a fistula formation.

**Figure 2:**
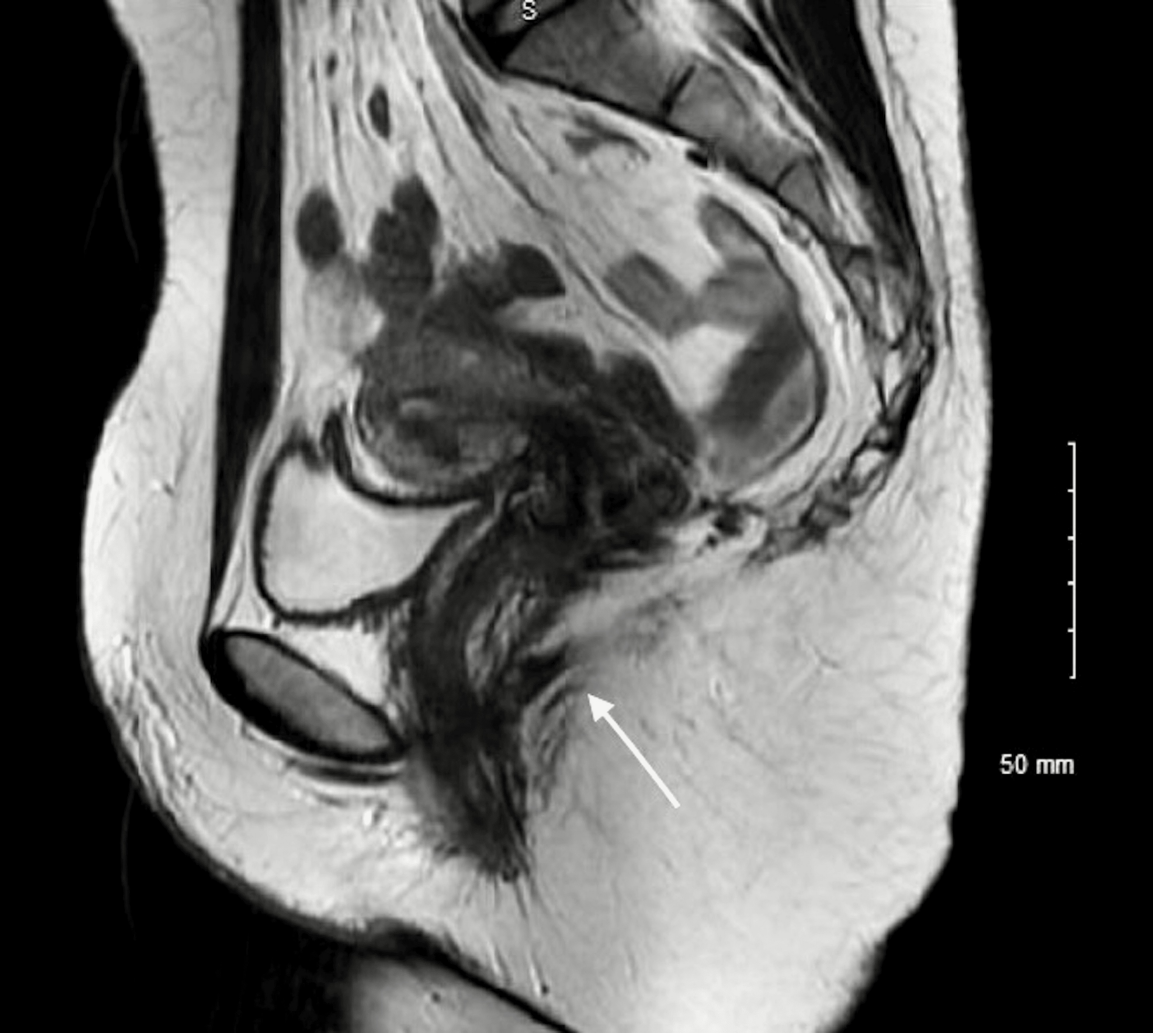
Pelvic MRI post-panproctocolectomy. Sagittal T2-weighted pelvic MRI demonstrates an enterovaginal fistula extending to the posterior vaginal vault. It was hypothesised that the fistula facilitates bacterial translocation into the vagina, with potential spread to the uterus, contributing to chronic endometritis. MRI: magnetic resonance imaging.

She also had recurrent bacterial vaginosis, initially responsive to metronidazole but prone to relapse. Magnetic resonance imaging (MRI) of the perineum revealed a linear high-signal tract extending posteriorly from the vaginal vault, consistent with a vaginal fistula. This was hypothesised to contribute to recurrent infections. A prolonged antibiotic regimen of ciprofloxacin and metronidazole was initiated. Repeat MRI confirmed a persistent fistula at the 6 o'clock position; however, enteroclysis showed no evidence of active or fibrotic CD or abscess formation (Figures 1, 2).

Remission of fistulising CD was maintained with adalimumab, azathioprine, and allopurinol. Additional symptoms included inflammatory arthralgia and morning stiffness affecting the elbows, knees, and hips, consistent with Crohn's-associated arthritis.

The couple began trying to conceive one year post-surgery without success after 12 months. She had no prior pregnancies or miscarriages (G0 P0). This prompted an evaluation for ART. Her BMI was 20, cycles were regular, and CD was medically stable. Ovarian reserve was favourable, with an antral follicle count (AFC) of 18 and an anti-Müllerian hormone (AMH) level of 16 pmol/L. Infection screens and cervical cytology were normal. Due to severe vitamin B12 deficiency, she received intramuscular B12 and folic acid.

The partner's sperm analysis showed oligoasthenospermia (low sperm count and motility). A DNA fragmentation test, which assesses the integrity of sperm DNA, demonstrated a very low fragmentation level (0.1%), indicating excellent genetic quality. Due to prolonged subfertility and male factor issues, intracytoplasmic sperm injection (ICSI), where a single sperm is injected into an egg, was performed.

In cycle one, thirteen good-quality oocytes were retrieved, yielding 10 fertilised embryos with ICSI. A single fully expanded blastocyst (day five, grade 4AA) was transferred, resulting in a positive biochemical pregnancy confirmed at four weeks' gestation, but a negative result at six weeks' gestation. Over the next two years, four additional ART cycles were performed, resulting in six embryo transfers (ETs) overall, but no further pregnancies occurred despite stable CD remission (Table 1).

**Table 1:** Summary of ART cycle. The patient underwent five ART cycles. Only one biochemical pregnancy occurred in the first cycle, ending in loss at gestation week six. High-quality blastocysts were obtained in multiple cycles, apart from one morula-stage embryo. FET: frozen embryo transfer; UPT: urine pregnancy test; MACS ICSI: magnetic-activated cell sorting with intracytoplasmic sperm injection; ART: assisted reproductive technology.

Cycle	Treatment	Oocytes	Fertilised	Transfer details	Outcome
1	ICSI	13	10	One blastocyst grade 4AA transferred day five	UPT positive at four weeks, negative at six weeks' gestation.
2	ICSI	10	7	One compacted morula grade A transferred day five	Negative UPT
3	ICSI	10	7	One blastocyst grade 4AA transferred day five	Negative UPT
4	MACS ICSI	14	13	Two blastocysts grade 4AA transferred day five	Negative UPT
5	Natural frozen embryo transfer (FET): one blastocyst thawed and used	-	-	One FET and one remained in storage	Negative UPT
6	Planned natural FET with luteal progesterone support	-	-	Cycle cancelled before embryo transfer	Cancelled

Between ART cycles two and three, further investigation identified group B Streptococcus (GBS) colonisation on endometrial microbiome analysis (EMMA) and analysis of infectious chronic endometritis (ALICE) panels. A biopsy confirmed heavy GBS colonisation. She was treated with metronidazole and co-trimoxazole (pending renal function), followed by lactobacillus-based probiotics to restore microbial balance and improve endometrial receptivity. During this period, she was also diagnosed with lichen sclerosus of the right vulva, which was managed with Fucibet and Dermol. Adalimumab was increased to weekly to maintain CD remission and minimise inflammation. Allopurinol was withheld two weeks prior to each ET and planned to remain paused during pregnancy to avoid teratogenic risk. Systemic corticosteroids were considered for vulvar CD but were not initiated to limit immunosuppression and infection risk before ET. Recurrent urinary tract infections (UTIs) between cycles were treated with nitrofurantoin to prevent ascending infection. Additional screening, including tests for sexually transmitted infections, thyroid function, vitamin D levels, full blood count, antiphospholipid antibodies, and vitamin B12, was performed to exclude other reversible factors that might impair implantation or pregnancy. After five unsuccessful ART cycles, the patient elected to discontinue treatment.

## Discussion

This report highlights the significant impact of CD surgery on ART live birth rates. It is a complex case of fistulising CD with chronic granulomatous inflammation involving the vulvovaginal region. Vulvar CD, a rare extraintestinal manifestation, typically presents with swelling and ulceration of the perineum [4]. Management is often challenging, and while anti-TNF therapy may lead to clinical improvement, relapse is common [5]. In our case, the patient was refractory to medication, and surgery was recommended. Her general condition improved postoperatively, but vulvovaginal symptoms persisted. Clinical remission was achieved with combined azathioprine and adalimumab, which is more effective than monotherapy. Newer targeted therapies such as ustekinumab and vedolizumab have shown promise, offering rapid and sustained anti-inflammatory effects for vulvar CD [5].

Fertility preservation remains a major concern for women with CD undergoing panproctocolectomy. It significantly reduces fertility compared to medical management. This effect is more pronounced in CD than in UC. A systematic review and meta-analysis by Laube et al. reported a 49-71% reduction in ART live birth rates in women with CD post IPAA [2]. In contrast, UC showed only a mild reduction (OR = 0.88, 95% CI: 0.67-1.17) [2]. Supporting this, a nationwide Danish cohort study of 539 women (381 with UC and 158 with CD) undergoing four or more ART cycles found significantly reduced live birth rates in CD patients post-surgery (adjusted OR: 0.29; 95% CI: 0.13-0.65), while UC patients were unaffected unless IPAA had failed (OR: 0.81; 95% CI: 0.47-1.40) [3].

Surgical interventions can cause tubal damage and pelvic adhesions [3]. While ART can bypass tubal infertility, pelvic adhesions from open surgery may still impair embryo implantation [3]. Laparoscopic procedures appear to preserve fertility better than open surgery. In a cohort of 50 women undergoing IPAA, only 11% of those who had laparoscopic surgery required ART to conceive, compared with 39% in the open surgery group. Additionally, the one-year pregnancy rate was higher after laparoscopy (56%) than after open surgery (30%), supporting the fertility-preserving advantages of laparoscopic IPAA [6].

However, pelvic adhesions alone do not fully explain the lower ART live birth rates seen in women with CD, as women with UC are not similarly affected [2,3]. Unlike UC, which is limited to the colon and can be cured by total proctocolectomy, CD is a transmural, relapsing disease that can involve any part of the gastrointestinal tract [6]. This suggests that infertility in CD may also be influenced by persistent systemic inflammation, immune dysregulation, and alterations in the endometrial microbiome following surgery. These mechanisms remain poorly understood and warrant further investigation.

Despite undergoing total proctocolectomy with definitive ileostomy (TPC-DI), CD recurrence remains common, reported in 4% of patients at one year, 27% at five years, and 39% at eight years, particularly among those with penetrating or perianal phenotypes (RR = 1.7; 95% CI: 1.5-1.9; p = 0.05) [7]. In our case, the patient experienced recurrence of vulvovaginal symptoms and VIN. Given the high relapse risk, medical therapy should be optimised and surgery deferred when feasible to preserve fertility, with counselling on potential outcomes [2,7]. In this instance, although the need for surgery was significant, preoperative counselling was limited.

Postoperative relapse can be reduced through early initiation of combination therapy with anti-TNF agents and immunomodulators, which improves treatment durability, minimises anti-drug antibody formation, and enhances mucosal healing [8]. Table 2 summarizes the possible contributing mechanisms of decreased infertility.

The timing of bowel surgery relative to ART may impact fertility. In a study of 86 CD and 121 UC patients, none of the 10 CD patients who underwent ART within two years conceived, whereas three of six UC patients did [6]. When ART was performed two to four years post-surgery, live births occurred in seven of 22 CD and 13 of 31 UC patients. Friedman et al. suggested that surgery within two years may impair fertility in CD due to inflammation, immune dysfunction, or adhesions, while UC appears less affected by timing [3]. Although our patient began ART two years after surgery and achieved only one biochemical pregnancy, outcomes are generally more favourable when ART is delayed beyond two years.

The extent of surgery also impacts fertility. A Swedish study of 415 women with CD found that those undergoing colectomy with rectal preservation (ileorectal anastomosis, IRA) had a hazard ratio (HR) for live birth of 0.67 (95% CI: 0.53-0.83, p < 0.001), while proctectomy led to a greater reduction (HR = 0.39, 95% CI: 0.30-0.52, p < 0.001) [7]. This suggests IRA may better preserve fertility when feasible [9]. In our case, proctectomy was necessary due to perianal and vulvar CD.

To maximise fertility outcomes, CD control and remission are essential [3]. Conception is more likely after at least six months of CD remission [10]. Active CD, particularly with perianal involvement, can impair ovarian reserve and implantation. One study reported an odds ratio of 27.9 (95% CI: 6.1-127.9, p < 0.001) for low AMH (<1.1 ng/mL;<7.9 pmol/L) in women with active disease [10]. In our case, AMH remained favourable (16 pmol/L) during remission, allowing retrieval of 10-13 oocytes. A meta-analysis of 418 CD patients found no link between surgery and AMH decline, emphasising the importance of maintaining remission [10].

Fistulas affect approximately 30% of patients with perianal CD, forming abnormal tracts between the bowel and adjacent structures. While they are common indications for surgery, fistulas may also develop postoperatively [11]. In our patient, an enterovaginal fistula developed after surgery. Infliximab is first-line therapy for fistulising CD, with adalimumab also effective. Combination therapy with adalimumab and ciprofloxacin has demonstrated better outcomes than monotherapy, consistent with our patient's management [11]. However, medical therapy does not fully close fistulas [11]. Although enteroclysis showed no abscess or active disease following treatment, repeat MRI confirmed a persistent fistula tract. The MDT hypothesised that recurrent UTIs, bacterial vaginosis, and endometritis were exacerbated by ongoing bacterial translocation into the vaginal vault. Fistula formation is a potential factor associated with microbiome dysbiosis and CE [11]. 

Studies on ART outcomes following Crohn's disease (CD) surgery do not clearly specify whether reduced live birth rates are associated with increased rates of recurrent implantation failure (RIF) or recurrent pregnancy loss (RPL) [3]. In the general population, RIF, defined as failure to conceive after three transfers of good-quality euploid blastocysts only affects approximately 5% of women undergoing IVF [12]. Contributing factors include age, BMI, stress, smoking, microbiome alterations, chronic endometritis (CE), and immunological or infectious causes [13,14]. Whether RIF rates increase after CD surgery remains unknown. However, a recent meta-analysis found that chronic endometritis (CE) is more strongly associated with RPL than with RIF. CE was identified in 37.6% of RPL cases compared with 16.4% of controls (OR: 3.59, 95% CI: 2.46-5.24, p<0.00001), whereas no significant association was observed with RIF (OR: 1.10, 95% CI: 0.26-4.61, p=0.90). Because CE is often asymptomatic and may go undetected, it could represent an important underlying cause of RPL in women with Crohn's disease [15].

Alterations in the microbiome have been linked to recurrent bacterial vaginosis and UTIs [13,14]. A healthy endometrium is typically dominated by Lactobacillus species, which support implantation. Low levels of Lactobacillus and increased pathogenic bacteria such as *Escherichia coli*, *Gardnerella vaginalis*, *Klebsiella pneumoniae*, and *Streptococcus* spp. are associated with reduced pregnancy outcomes [13,14]. Bacterial pathogens can disrupt the endometrial microbiota, increasing the risk of CE [13,14]. In our patient, group B Streptococcus was identified. Diagnosis of CE relies on endometrial biopsy with CD138 immunostaining to detect plasma cells, while molecular tools such as EMMA and ALICE can assess microbiome composition [14]. The patient was treated with metronidazole and co-trimoxazole, followed by Lactobacillus-based probiotics to restore microbial balance. Standard treatment for CE involves oral antibiotic administration (OAA), with reported success rates ranging from 58.95% to 99.15% [16]. Combining OAA with intrauterine antibiotic infusion (IAI) further improves effectiveness [16]. IVF success rate improves only after confirmed eradication of CE on repeat biopsy [16-18] (Table 2).

**Table 2:** Mechanisms of decreased ART live birth rates following Crohn's disease surgery. Possible mechanisms contributing to decreased ART live birth rates in Crohn's disease following panproctocolectomy. *Active inflammation, chronic endometritis with endometrial dysbiosis, and immunological imbalance may further impair ART outcomes after surgery, although these factors are not directly caused by the surgery itself. ART: assisted reproductive technology, AMH: anti-Müllerian hormone, IVF: in vitro fertilisation, CD: Crohn's disease.

Mechanism	Details
Anatomical factors	Pelvic adhesions may impair implantation [3].Laparoscopic surgery is more fertility-preserving than open surgery and associated with higher ART live birth rates than. Tubal damage can be overcome by IVF [6].
Timing and extent of surgery	ART outcomes are significantly poorer when performed within two years of major bowel surgery [3,6]. Colectomy and proctectomy increase infertility. Rectal preservation and ileorectal anastomosis in CD improve fertility outcomes [7].
Vitamin B12 deficiency and homocysteine elevation	End ileal resection causes vitamin B12 deficiency, leading to elevated homocysteine, which can impair oocyte and embryo quality, disrupt implantation, and increase miscarriage risk [19,20].
*Active inflammation and disease activity	Persistent systemic inflammation impairs implantation and lowers ovarian reserve (AMH) [3,10].
*Chronic endometritis (CE) and endometrial dysbiosis	Microbiome imbalance can reduce endometrial receptivity and implantation. Chronic endometritis (CE) is linked to higher rates of recurrent pregnancy loss (RPL). In addition, bacterial translocation through vaginal fistulas may lead to infections that disrupt the local microbiome and promote the development of CE, further compromising fertility [13,14].
*Immunological Imbalance (↑ Th1 cytokines, ↑ TNF-α)	Suppresses trophoblast growth and induces pro-inflammatory and thrombotic changes in uterine vessels, thereby impairing placentation and implantation [13,14,17].

Immunological imbalance has also been linked to RIF, particularly through the Th1/Th2 cytokine shift influencing endometrial receptivity [13,14]. Elevated Th1 cytokines, especially TNF-α, can suppress trophoblast growth and trigger pro-inflammatory and thrombotic changes in the uterine vasculature, impairing implantation [13,14]. TNF-α levels rise throughout pregnancy, and elevated levels are associated with miscarriage, foetal loss, pre‑eclampsia, and preterm birth [17]. In contrast, Th2 cytokines (IL-4, IL-6, IL-10) counterbalance Th1-mediated inflammation. Women with RIF often show high TNF-α/IL-4 and TNF-α/IL-10 ratios, indicating a pro-inflammatory shift [13]. Because CD is characterised by a Th1-dominant immune profile, which may persist after TPC-DI due to relapse, immunological imbalance may contribute to RIF following surgery [7]. Anti-TNF therapies (e.g., infliximab, adalimumab) promote mucosal healing in inflammatory bowel disease and reduce corticosteroid use, maintaining CD remission [2,18]. Meta-analyses and cohort studies suggest that women with medically controlled CD, especially with anti-TNF therapy, have IVF outcomes comparable to the general population [2,4]. Therefore, anti-TNF therapy may support implantation and pregnancy in women with IBD by improving the underlying inflammatory environment.

Patients with CD are also at increased risk of vitamin B12 (cobalamin) deficiency, particularly when the terminal ileum is affected by inflammation or has been surgically resected. In a cohort of 381 surgical CD patients, 33% were found to be B12-deficient when assessed using holotranscobalamin and methylmalonic acid. Key risk factors included ileal resection ≤20 cm (OR: 3.0, 95% CI: 1.5-6.0), resection >20 cm (OR: 6.7, 95% CI: 3.0-14.7), and active ileal inflammation (OR: 3.9, 95% CI: 2.2-6.9), all statistically significant (P < 0.01) [19]. Vitamin B12, a cofactor for folate-dependent methionine synthase, is essential for the remethylation of homocysteine and plays a vital role in conception. Vitamin B12 deficiency can lead to elevated homocysteine levels, which increase oxidative stress and impair ovulation, oocyte maturation, and embryo development. A Dutch study reported a positive correlation between serum and follicular fluid concentrations of vitamin B12 and embryo quality [20]. Elevated homocysteine may also damage the endometrium, resulting in defective implantation. Additionally, it can induce apoptosis and impair trophoblastic function, thereby compromising placental development and increasing the risk of spontaneous abortion. It is therefore essential to supplement vitamin B12 and folate, as in our patient, with intramuscular B12 administered prior to ART. Overall, higher serum concentrations of folate and vitamin B12 improve oocyte and embryo quality, enhance implantation, and increase live birth rates [20]. Table 3 summarises possible interventions to improve fertility outcomes.

**Table 3:** Strategies to improve ART live birth rates in Crohn's disease. Strategies to improve ART live birth rates in women with refractory Crohn's disease include optimising medical therapy and carefully timing surgical intervention. Additional focus on endometrial and immunological factors may further enhance fertility outcomes. ART: assisted reproductive technology, CD: Crohn's disease, TNF-α: tumour necrosis factor-alpha, IPAA: ileal pouch-anal anastomosis.

Intervention	Recommendation
Medical therapy	Optimise medical therapy before resorting to surgery to preserve fertility. Combination therapy (e.g., azathioprine + adalimumab) which is more effective than monotherapy [11].
Preoperative counselling	Counselling on fertility preservation and surgical risks should be conducted prior to surgical planning [2].
Surgical timing	ART should be delayed for at least two years following bowel surgery due to lower success rates with earlier attempts [6].
Surgical approach	Laparoscopic IPAA is preferred over open surgery due to reduced postoperative adhesions and improved fertility outcomes [6].
Surgery type	Colectomy without proctectomy and with ileorectal anastomosis (IRA) is more fertility-preserving than pan proctocolectomy. Rectal preservation should be considered when feasible [7].
Postoperative management	Initiate anti-TNF treatment early post-surgery to prevent recurrence. Achieve ≥6 months remission prior to ART. Active CD reduces ovarian reserve. Treat fistulising CD with infliximab or adalimumab + ciprofloxacin [8-11].
Endometrial environment	Assess for chronic endometritis (CE) and microbiome dysbiosis. Treat with targeted antibiotics and probiotics to restore endometrial receptivity [13,14].
Immune modulation	Address Th1/Th2 imbalance; Th1 cytokines (e.g., TNF-α) impair implantation. Use anti-TNF agents (infliximab, adalimumab) to reduce inflammation and improve endometrial receptivity [13-18].
Nutritional optimisation	Supplement vitamin B12 and folate to reduce homocysteine levels. Improves oocyte and embryo quality and enhances endometrial receptivity [19,20].

## Conclusions

Major abdominal surgery for CD appears to be associated with lower ART live birth rates, even during remission. This likely involves multiple factors, including pelvic adhesions, surgical timing and extent, chronic endometritis, disruption of the microbiome, and immune imbalance. Nutritional consequences of surgery, such as vitamin B12 deficiency and hyperhomocysteinaemia after terminal ileal resection, may impair oocyte quality, implantation and placentation. Persistent fistulas can allow bacterial translocation, causing recurrent vaginosis and CE, while endometrial dysbiosis and a Th1/TNF-α-dominant immune state may further hinder implantation. Laparoscopic procedures may mitigate risks from pelvic adhesions compared to open surgery. Early use of targeted therapies and immunomodulators post-surgery could improve endometrial receptivity. These observations are hypothesised and should be interpreted within the limitations of a single case report, with further research needed to confirm the proposed associations between CD surgery and reduced fertility. This case emphasises the need for a multidisciplinary approach to disease control, nutrition, preoperative fertility counselling, and surgical planning. It highlights the importance of new guidelines to preserve fertility in women of reproductive age undergoing surgical management for CD.

## References

[1] Lungaro L, Costanzini A, Manza F (2023). Impact of female gender in inflammatory bowel diseases: a narrative review. J Pers Med.

[2] Laube R, Tran Y, Paramsothy S, Leong RW (2021). Assisted reproductive technology in Crohn's disease and ulcerative colitis: a systematic review and meta-analysis. Am J Gastroenterol.

[3] Friedman S, Larsen PV, Fedder J, Nørgård BM (2017). The efficacy of assisted reproduction in women with inflammatory bowel disease and the impact of surgery—a nationwide cohort study. Inflamm Bowel Dis.

[4] Kyriakou G, Gkermpesi M, Thomopoulos K, Marangos M, Georgiou S (2019). Metastatic vulvar Crohn's disease preceding intestinal manifestations: a case report and short review. Acta Dermatovenerol Alp Pannonica Adriat.

[5] Cho JM, Loftus EV Jr, Bruining DH (2021). Vulvar Crohn's disease: clinical features and outcomes. Am J Gastroenterol.

[6] Bartels SA, D’Hoore A, Cuesta MA (2012). Significantly increased pregnancy rates after laparoscopic restorative proctocolectomy: a cross-sectional study. Ann Surg.

[7] Amiot A, Gornet JM, Baudry C (2011). Crohn's disease recurrence after total proctocolectomy with definitive ileostomy. Dig Liver Dis.

[8] Bachour SP, Click BH (2024). Clinical update on the prevention and management of postoperative Crohn's disease recurrence. Curr Gastroenterol Rep.

[9] Druvefors E, Myrelid P, Andersson RE, Landerholm K (2024). Female and male fertility after colectomy and reconstructive surgery in inflammatory bowel disease: a national cohort study from Sweden. J Crohns Colitis.

[10] Foulon A, Richard N, Guichard C (2024). Factors associated with decreased ovarian reserve in Crohn's disease: a systematic review and meta-analysis. Acta Obstet Gynecol Scand.

[11] Wetwittayakhlang P, Al Khoury A, Hahn GD, Lakatos PL (2022). The optimal management of fistulizing Crohn's disease: evidence beyond randomized clinical trials. J Clin Med.

[12] Pirtea P, Cedars MI, Devine K (2023). Recurrent implantation failure: reality or a statistical mirage?: consensus statement from the July 1, 2022 Lugano Workshop on recurrent implantation failure. Fertil Steril.

[13] Bashiri A, Halper KI, Orvieto R (2018). Recurrent implantation failure-update overview on etiology, diagnosis, treatment and future directions. Reprod Biol Endocrinol.

[14] Ma J, Gao W, Li D (2022). Recurrent implantation failure: a comprehensive summary from etiology to treatment. Front Endocrinol (Lausanne).

[15] Ticconi C, Inversetti A, Marraffa S, Campagnolo L, Arthur J, Zambella E, Di Simone N (2024). Chronic endometritis and recurrent reproductive failure: a systematic review and meta-analysis. Front Immunol.

[16] Cheng X, Huang Z, Xiao Z, Bai Y (2022). Does antibiotic therapy for chronic endometritis improve clinical outcomes of patients with recurrent implantation failure in subsequent IVF cycles? A systematic review and meta-analysis. J Assist Reprod Genet.

[17] Alijotas-Reig J, Esteve-Valverde E, Ferrer-Oliveras R, Llurba E, Gris JM (2017). Tumor necrosis factor-alpha and pregnancy: focus on biologics. An updated and comprehensive review. Clin Rev Allergy Immunol.

[18] Ungar B, Levy I, Yavne Y (2016). Optimizing anti-TNF-α therapy: serum levels of infliximab and adalimumab are associated with mucosal healing in patients with inflammatory bowel diseases. Clin Gastroenterol Hepatol.

[19] Ward MG, Kariyawasam VC, Mogan SB (2015). Prevalence and risk factors for functional vitamin B12 deficiency in patients with Crohn's disease. Inflamm Bowel Dis.

[20] Gaskins AJ, Chiu YH, Williams PL, Ford JB, Toth TL, Hauser R, Chavarro JE (2015). Association between serum folate and vitamin B-12 and outcomes of assisted reproductive technologies. Am J Clin Nutr.

